# Variability of multi-omics profiles in a population-based child cohort

**DOI:** 10.1186/s12916-021-02027-z

**Published:** 2021-07-22

**Authors:** Marta Gallego-Paüls, Carles Hernández-Ferrer, Mariona Bustamante, Xavier Basagaña, Jose Barrera-Gómez, Chung-Ho E. Lau, Alexandros P. Siskos, Marta Vives-Usano, Carlos Ruiz-Arenas, John Wright, Remy Slama, Barbara Heude, Maribel Casas, Regina Grazuleviciene, Leda Chatzi, Eva Borràs, Eduard Sabidó, Ángel Carracedo, Xavier Estivill, Jose Urquiza, Muireann Coen, Hector C. Keun, Juan R. González, Martine Vrijheid, Léa Maitre

**Affiliations:** 1 ISGlobal, Barcelona, Spain; 2 Universitat Pompeu Fabra (UPF), Barcelona, Spain; 3 Consorcio de Investigacion Biomedica en Red de Epidemiologia y Salud Publica (CIBERESP), Madrid, Spain; 4 Center for Genomic Regulation (CRG), Barcelona Institute of Science and Technology (BIST), Barcelona, Spain; 5 MRC Centre for Environment and Health, School of Public Health, Imperial College London, London, UK; 6 Division of Systems Medicine, Department of Metabolism, Digestion and Reproduction, Imperial College London, South Kensington, London, UK; 7 Cancer Metabolism & Systems Toxicology Group, Division of Cancer, Department of Surgery & Cancer and Division of Systems Medicine, Department of Metabolism, Digestion & Reproduction, Imperial College London, London, UK; 8 Bradford Institute for Health Research, Bradford Teaching Hospitals NHS Foundation Trust, Bradford, UK; 9 Team of Environmental Epidemiology applied to Reproduction and Respiratory Health, Institute for Advanced Biosciences (IAB), Inserm, CNRS, Université Grenoble Alpes, Grenoble, France; 10 Université de Paris, Centre for Research in Epidemiology and Statistics (CRESS), INSERM, INRAE, F-75004 Paris, France; 11 Department of Environmental Sciences, Vytautas Magnus University, Kaunas, Lithuania; 12 Department of Preventive Medicine, Keck School of Medicine, University of Southern California, Los Angeles, CA USA; 13 Medicine Genomics Group, Centro de Investigación Biomédica en Red Enfermedades Raras (CIBERER), University of Santiago de Compostela, CEGEN-PRB3, Santiago de Compostela, Spain; 14 Galician Foundation of Genomic Medicine, Instituto de Investigación Sanitaria de Santiago de Compostela (IDIS), Servicio Gallego de Salud (SERGAS), Santiago de Compostela, Galicia Spain; 15 Oncology Safety, Clinical Pharmacology and Safety Sciences, R&D, AstraZeneca, Cambridge, UK

**Keywords:** Multi-omics, Exposome, Variability, Population study, Metabolomics, DNA methylation, Cross-omics, mRNA, miRNA, Children

## Abstract

**Background:**

Multiple omics technologies are increasingly applied to detect early, subtle molecular responses to environmental stressors for future disease risk prevention. However, there is an urgent need for further evaluation of stability and variability of omics profiles in healthy individuals, especially during childhood.

**Methods:**

We aimed to estimate intra-, inter-individual and cohort variability of multi-omics profiles (blood DNA methylation, gene expression, miRNA, proteins and serum and urine metabolites) measured 6 months apart in 156 healthy children from five European countries. We further performed a multi-omics network analysis to establish clusters of co-varying omics features and assessed the contribution of key variables (including biological traits and sample collection parameters) to omics variability.

**Results:**

All omics displayed a large range of intra- and inter-individual variability depending on each omics feature, although all presented a highest median intra-individual variability. DNA methylation was the most stable profile (median 37.6% inter-individual variability) while gene expression was the least stable (6.6%). Among the least stable features, we identified 1% cross-omics co-variation between CpGs and metabolites (e.g. glucose and CpGs related to obesity and type 2 diabetes). Explanatory variables, including age and body mass index (BMI), explained up to 9% of serum metabolite variability.

**Conclusions:**

Methylation and targeted serum metabolomics are the most reliable omics to implement in single time-point measurements in large cross-sectional studies. In the case of metabolomics, sample collection and individual traits (e.g. BMI) are important parameters to control for improved comparability, at the study design or analysis stage. This study will be valuable for the design and interpretation of epidemiological studies that aim to link omics signatures to disease, environmental exposures, or both.

**Supplementary Information:**

The online version contains supplementary material available at 10.1186/s12916-021-02027-z.

## Background

Characterizing early indicators of health and disease trajectories during pregnancy and childhood is at the core of the life course approach [1–4]. Early life englobes the most critical/sensitive periods for organ development, which makes it especially vulnerable to the effects of environmental exposures [5, 6]. The integration of multiple omics data—such as epigenomics, transcriptomics, proteomics and metabolomics—is increasingly applied to detect early, subtle molecular responses to environmental exposures because it employs a holistic view on all cellular processes [7–10]. However, there is an urgent need for further evaluation of stability and variability of omics profiles, between and within healthy children. Epidemiological studies that incorporate omics profiles to monitor healthy individuals over time need to be informed of technical and biological variability in order to interpret changes in omics profiles, even if they are small. Omics variability may be determined by factors hindering subtle biological changes of interest, such as seasonality, individual characteristics (age and BMI), stage of life (i.e. hormones might vary between pre-puberty and adulthood stages) as well as by technical variability (due to measurement error and limited precision of analytic tools), which therefore must be controlled at the design of the study [2, 5, 6].

Temporal variability in omics profiles has been described previously to assess the reliability of single time-point measurements in cross-sectional studies and to understand ageing and disease processes [11, 12]. In this paper, we define “intra-individual variability” as the variability estimated within individuals over time and “inter-individual variability” as the between-individuals variability. We also define the “short-term” as a time span of hours or days, the “medium-term” as a time span of months, and the “long-term” when considering years. For example, in the short term, the metabolome in urine and blood is assumed to be more dynamic than other omics as it is the downstream result of in vivo substances and environmental factor influence [4]. On the other hand, DNA methylation is generally considered to be the most stable omics profile over short periods of time [13] and could provide more valuable information for environmental epidemiological purposes than other omics, as some methylomics signatures (e.g. smoking signatures) have been shown to persist over time even when the exposure no longer exists [2, 13–19]. Previous studies have also shown low levels of intra-individual variability in > 95% of the gene expression profile and in 25.5% of the miRNAs analysed, which are proposed as good biomarkers for many human diseases [20–27]. Overall, the blood proteome is considered quite stable over time with strong inter-individual variability due to genetics, although some proteins are highly influenced by body mass composition and acute inflammation [28].

To date, multi-omics platforms have mainly been used in studies with a small sample size that focused on dietary or physical activity interventions rather than following up healthy people from the general population [2, 29, 30]. They agreed that inter-individual variation, rather than intra-individual variation, was the main explanatory factor for all omics measurements. However, previous multi-omics profile studies have not considered changes related to short and medium term. Especially, there is a lack of evidence regarding children from the general population, nor single or multi-omics studies, and the contribution of several factors such as age, sex and BMI.

**Fig. 1:**
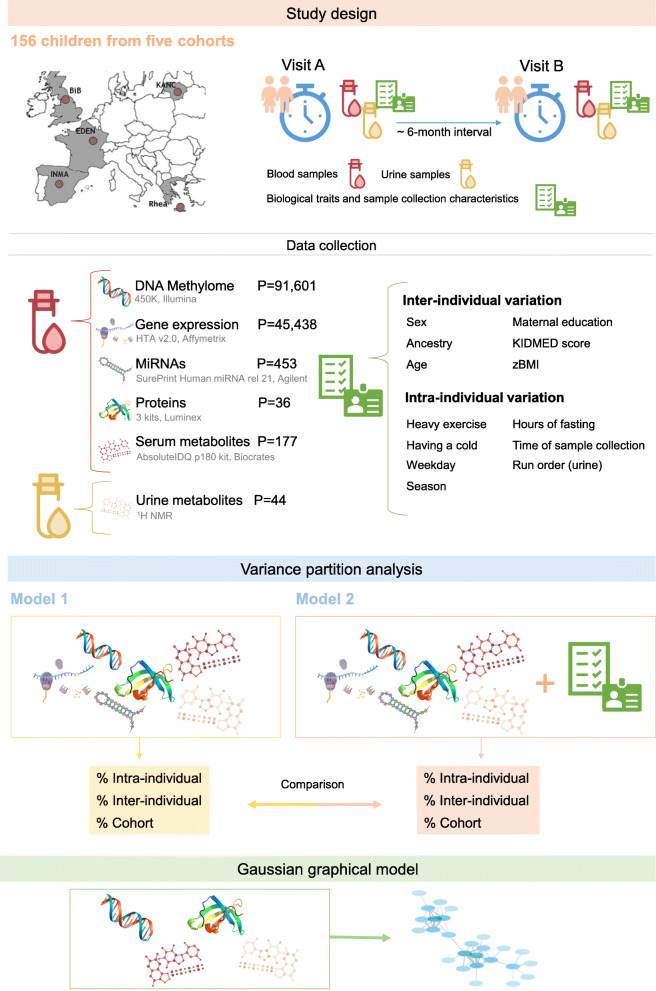
Study workflow

In the present study, we estimated intra- and inter-individual variability in multi-omics profiles (blood DNA methylation, gene expression, miRNA, proteins and serum and urine metabolites) in 156 children from five European countries at two time points with a 6-month interval. We further aimed to assess interrelationships between the variability in different omics layers by performing a cross-omics correlation network analysis. Finally, we aimed to decompose the variance in multi-omics profiles according to (1) inter-individual characteristics: sex, ancestry, age, maternal education, Mediterranean diet quality index (KIDMED score) and zBMI (which do not change in a 6-month period); and (2) intra-individual characteristics: hours of fasting before the visit for blood/ urine sampling, heavy exercise practice the same day or the day before sampling, having a cold at the moment of the visit, hour of sampling, day of the week and season (Fig. 1).

## Methods

### Study design and population

The HELIX (Human Early Life Exposome) study is a collaborative project of six-population based cohorts in different European Countries: UK (BiB: Born in Bradford) [31], France (EDEN: Étude des Déterminants pré et postnatals du développement et de la santé de l’Énfant) [32], Spain (INMA: Infancia y Medio Ambiente) [33], Lithuania (KANC: Kaunus cohort) [34], Norway (MoBa: Norwegian Mother and Child Cohort Study) [35] and Greece (RHEA: Mother Child Cohort study in Crete) [6, 36]. The aim of the HELIX study was to measure and describe multiple environmental exposures during early life (pregnancy and childhood) in a prospective cohort and associate these exposures with molecular omics signatures and child health outcomes.

From the six existing European longitudinal population-based birth cohorts studies participating in HELIX, a subcohort of 1301 mother-child pairs was selected to be fully characterized for a broad suite of environmental exposures and omics data, to be clinically examined, and to have biological samples collected [6]. From this subcohort, 156 children were selected to be part of the panel study: 28 from BiB (UK), 28 from EDEN (France), 40 from INMA Sabadell (Spain), 30 from KANC (Lithuania) and 30 from RHEA (Greece) [37]. Children from MoBa (Norwegian Mother and Child Cohort Study; Norway) were not included in this panel study. The Child Panel Study had the same inclusion criteria as the HELIX subcohort: (a) age 6–11 years at the time of the visit, with a preference for ages 7–9 years if possible; (b) sufficient stored pregnancy blood and urine samples; (c) complete address history available; and (d) no serious health problem.

Concretely, the panel study consisted of two visits (A and B) where data on exposures, individual behaviours, phenotypes and omics profiles were collected [38]. The mean difference between the two visits (A and B) was 6.11 months (standard deviation (SD): 2.18 months). In order to characterize in depth the variability of the omics measurements, the study population was further restricted to children with complete information for both visits (A and B) for at least one of the omics (N=156).

Prior to the start of data collection, national ethics committees had granted all the required permissions that allowed cohort participant recruitment and follow-up visits. Additionally, all the participants were asked to sign a HELIX specific informed consent.

### Sample collection

Biological samples were collected using the same standardized protocols across all five cohorts. Urine samples were collected twice daily (first morning void and bedtime sample) in high-quality polypropylene tubes. The two urine samples were brought by the participants to the centre in cool packs and stored at − 4 °C until processing. After aliquoting, the urine samples were frozen at − 80 °C under optimized and standardized procedures. A pooled sample of both the morning and the night urine samples was used for the analysis when available (94.9% of individuals in the first visit and 95.5% in the second visit). In visit A, 7 children only had a morning sample available, and 1 child only had night sample. For visit B, this happened in 4 and 3 children for morning and night samples, respectively.

Eighteen milliliters of blood was collected at the end of the clinical examination of the child, ensuring an approximate 3-h fasting time since the last meal (visit A mean: 3:34 h (SD: 1:11 h); visit B mean: 2:35 h (SD: 1:31 h)). Blood samples were collected using a ‘butterfly’ vacuum clip and local anaesthetic and processed into a variety of sample matrices for serum, plasma, whole blood for RNA extraction, red cells and a buffy coat for DNA extraction. After processing, these samples were frozen at − 80 °C under optimized and standardized procedures [6].

### Summary of laboratory processing of omics signatures, quality control and normalization

We performed in-depth omics profiling at two time points ~ 6 months apart for all 156 children. Because only 87 children had complete data of all omics analyses at both visits, we decided to analyse each omics profile independently (i.e. all paired samples available for each specific omics layer). The final sample size for each omics layer was 149, 105, 100, 149, 154 and 154 children for DNA methylation, blood gene expression, miRNA expression, proteins, serum metabolites and urine metabolites, respectively. Details on laboratory and data processing methods are available in Additional file 1 - Supplementary Methods [39–48]. While DNA methylation, gene and miRNA expression screenings were based on genome-wide arrays, the other methods were targeted or semi-targeted. From now on, we use the term “features” to refer to the omics variables in our study: CpGs, gene and miRNA transcripts, proteins and metabolites. Because our study did not have technical replicates (biological samples systematically aliquoted in two replicates before sample preparation), which would be the ideal way to measure technical variability, potential technical variability was filtered out as much as possible in each omics layers, before fitting the variance partition models.

All samples were randomized in the arrays by sex and cohort, and in addition, the samples from the same individual in the microarray-based platforms were paired in the same plate/array (see Supplementary Methods). In the methylation, gene and miRNA expression data, we corrected remaining technical batch effects and blood cell composition by calculating surrogate variables (SVs) while protecting for cohort, sex and age with the SVA and SmartSVA methods [44, 49]. We used residuals from the correction process to analyse blood DNA methylation, gene and miRNA expression. We excluded probes for CpGs that did not reach a 62.5% interclass correlation coefficient (ICC) to minimize technical variability, based on a previous analysis with technical replicates [46]. Gene and miRNA expression were filtered out based on call rate or other omics based on other technical parameters (see Table 1). All omics measurements were normalized and log2 transformed except for DNA methylation.

**Table 1:** Omics data description and technical variability management

Omics profile	Matrix	Sample size (omics available for both visits)	Number of features	Laboratory processing	Batch correction	Criteria for feature exclusion
**DNA methylation**	Blood leukocytes	149	91601	Randomized by cohort and sex, and panel samples paired in plate and array	Residuals of SVs protecting for cohort, sex and age. Cell type composition also corrected with SVs.	< 98% call rate and < 62.5% ICC
**Gene expression**	Whole blood	105	45438	Randomized by cohort and sex, and panel samples paired in plate.	Residuals of SVs protecting for cohort, sex and age. Cell type composition also corrected with SVs.	< 25% call rate
**miRNA expression**	Whole blood	100	453	Randomized by cohort and sex, and panel samples paired in plate and array.	Residuals of SVs protecting for cohort, sex and age. Cell type composition also corrected with SVs.	< 25% call rate
**Proteins**	Plasma	149	36	Randomized by cohort	Overall protein average minus plate specific protein average subtracted for each individual and each protein	< 30% measurements in the linear range (LIN)
**Serum metabolites**	Serum	154	177	Fully randomized	-	> 30% CV and > 30% BLD + zeros
**Urine metabolites**	Urine	154	44	Fully randomized	-	> 30% CV

Definitions. Call rate (for DNA methylome): proportion of detection of a given CpG among samples; Call rate (for miRNA and gene expression): proportion of detection of gene or miRNA among samples. Abbreviations. *SV* surrogate variables, *ICC* interclass correlation coefficient [35], *CV* coefficient of variation, *BLD* below limit of detection

### Statistical analyses

All statistical analyses were performed using R version 3.6.3 [50].

### Linear mixed effect models

Variability present in the different omics layers was calculated with the *variancePartition* R package [47]. Briefly, it fits a linear mixed effect model to partition the variance attributed to multiple variables in the data. As this analytical process uses a multiple regression model, the effect of each variable is assessed while correcting for the others. Therefore, *variancePartition* assessed the contribution of each meta-data variable to variation in each feature.

We considered two mixed effect models: (1) a model to estimate the proportion of variance attributable to intra-individual, inter-individual and cohort variability and (2) the same model adjusting for several explanatory variables (inter- and intra-individual variability-related variables, see list below) to determine the proportion of variance they accounted for. Individual IDs were entered in the models to account for inter-individual variability, whereas we took residuals as a measure of intra-individual variability.

The following explanatory variables were added to the model as a measure of (1) inter-individual variation: sex, ancestry of the child, age, maternal education—as a general measure of socio-economic status—KIDMED score as a measure of healthy diet pattern [51] and zBMI, as we did not observe significant changes in the 6-month period; and of (2) intra-individual variation: time to last meal (hours of fasting), heavy exercise practice the same day or the day before the sample collection, having a cold at the moment of sampling, hour of sample collection, run order for the urine metabolome model, day of the week and season at which the samples were collected. These were biological traits and sample collection parameters that were obtained through questionnaires. All omics, except the urine metabolome, were corrected for omics platform technical variables; therefore, we only included the run order as a covariate in the urine metabolome model. Time to last meal and hour of sample collection were not included in the urine metabolome model because we used pooled samples (morning and night).

Before running the models, we ensured the absence of collinearity between the explanatory variables by obtaining a collinearity score: if this score were to be > 0.99, the variance partition estimates would produce misleading results and overestimate the contribution of variables modelled as fixed effects [47]. No variables were eliminated due to collinearity.

In the case of the methylome, we aimed to assess the amount of variance attributed to differences in the immunological cell type composition. For this purpose, an extra model was performed with a dataset corrected for batch effect using the ComBat method [52] instead of using SVA method, in order to keep the effect of cell type composition.

Additionally for the methylome, we checked whether CpGs located in the 4th quartile for intra- and inter-individual variability were enriched for CpG island relative position (island, shore, shelf, open sea) and for overlap with CpGs associated with exposures/traits in the EWAS Atlas [53].

### Gaussian graphical modelling (GGM)—network analysis

A GGM was used to assess direct associations between changes in omics features and elucidate biologically relevant associations [54–58]. GGMs were built on the delta matrix calculated as the change in omics features between visits (i.e. the change in feature X for a child between time points is correlated with the change in Y, where the correlation is again calculated across all children). The omics features included: CpG sites with 100% intra-individual variability and proteins, serum and urine metabolites in the highest quartile for intra-individual variability. Gene expression and miRNAs were excluded because the number of participants with complete data including these layers was significantly lower (n=87) and would penalize the identification of biologically relevant associations. The data matrix contained 139 samples × 13,167 features (13,103 CpGs, 9 proteins, 44 serum metabolites and 11 urine metabolites). We computed GGMs using the *ggm.estimator.pcor* function from the R package “GeneNet” [48]. This function estimates pairwise partial correlation coefficients conditioned against all remaining variables, allowing to filter out indirect associations that may appear in omics data [48, 54]. We considered significant partial correlations between features those with *p* values below the false discovery rate (FDR) threshold of 0.05 (*p <* 1.28 × 10−7). To construct and visualize the resulting GGM networks, we used Cytoscape 3.8.2 [59]. Edges connecting the nodes represent significant partial correlations and they were weighted using partial correlation coefficients (PCCs). The opacity of each node is based on its connectivity degree (number of edges connecting a particular feature).

## Results

### Study participants

A total of 156 children from five cohorts across Europe (BIB in the UK, EDEN in France, KANC in Lithuania, RHEA in Greece and INMA in Spain) were followed up for this study, with demographic data detailed in Table 2. Time span between both visits (A and B) was 6.1 months (2.2 SD). Children were on average 7.8 (1.7 SD) years old in visit A. Most of the participants of the study (71.2%) were in the healthy BMI range and remained in the same category from visit A to visit B. Samples were collected on average 3:36 h (1:12 SD) after the last meal during visit A, and after 2:36 h (1:30 SD) during visit B. The hour of sample collection at both visits was almost the same, being 16:54 h (2:54 SD) and 16:18 h (3:06 SD) for visits A and B, respectively. Table 2 shows the description of other explanatory variables measured in the study.

**Table 2:** Population description (N=156)

		Start of the study
**Sex**	Male	89
	Female	67
**Ancestry**	European ancestry	145
	Pakistani	10
	Other	1
**Cohort**	BIB, UK	28
	EDEN, France	28
	KANC, Lithuania	30
	RHEA, Greece	30
	INMA, Spain	40
**Age** (years); mean (SD)	Total	7.8 (1.7)
	BIB	6.7 (0.2)
	EDEN	10.8 (0.5)
	KANC	6.7 (0.5)
	RHEA	6.3 (0.12)
	INMA	8.6 (0.5)
**zBMI;** mean (SD)		0.4 (1.2)
**zBMI categories**	Thinness (zBMI < − 2)	1
	Healthy (− 2 ≤ zBMI < 1)	111
	Overweight (1 ≤ zBMI ≤ 2)	27
	Obese (zBMI > 2)	17

### Variance partition analysis shows large heterogeneity between and within omics

**Fig. 2:**
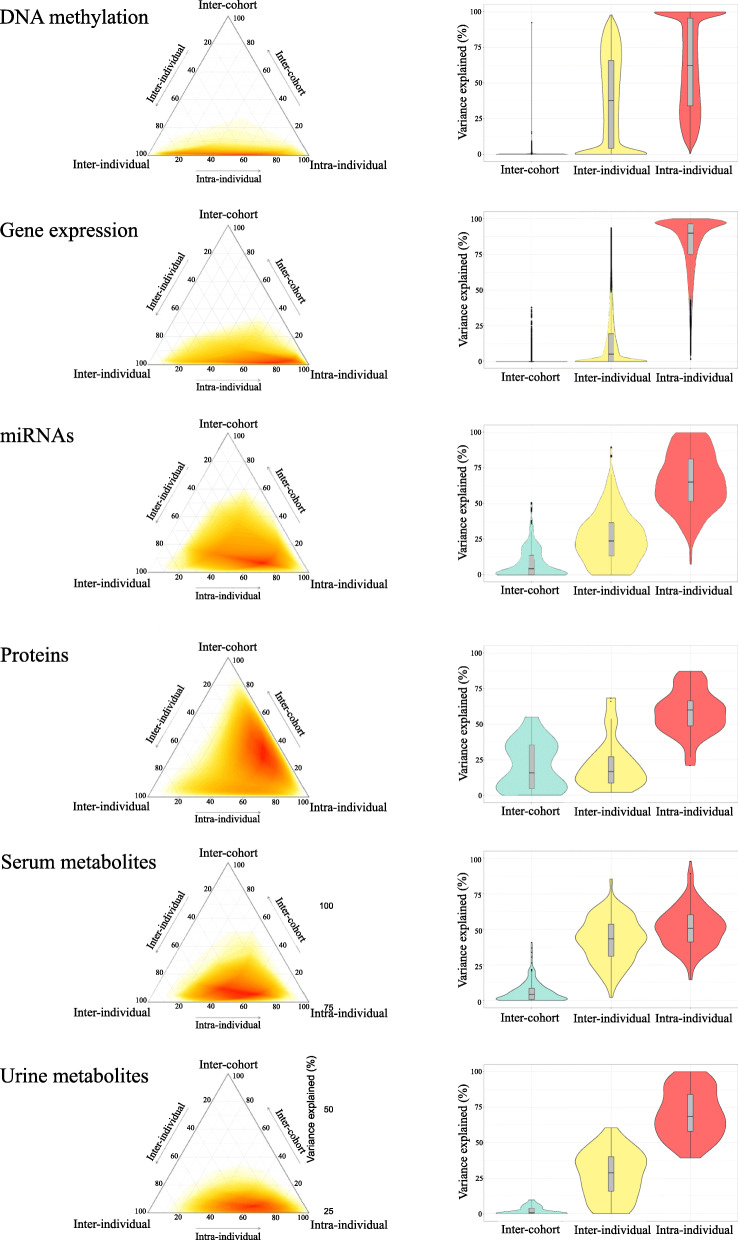
Variance partition analysis of omics data. Total variance was apportioned between cohort, inter-individual and intra-individual effects. **A** The heatmap colour (yellow to red) indicates the variance of features at each coordinate. **B** The violin plot describes the statistics of the variance explained by each component

A large heterogeneity in terms of the proportion of variance explained by cohort, inter- and intra-individual variability between omics layers and within the same omics layer was found (Fig. 2 and Table S1). Overall, omics features presented little variability due to cohort (median variability of features ranging from 0% for the methylome to 15.7% for the proteome).

### DNA methylation

Variation in blood DNA methylation was mainly due to intra-individual variability with a median of 62.2% across all CpG sites and a median inter-individual variability of 37.6% (Q1: 4.1%; Q3: 65.8%). We note however a large heterogeneity between the CpGs with the lower and upper quartiles ranging from 33.9 to 95.6% for intra-individual variability and from 4.1 to 65.8%, for inter. This heterogeneity was partially expected due to the intra-experimental variation in Illumina BeadChip data. Indeed, intra-individual variability of each CpG site in our study was associated to previously reported ICCs, described in the same tissue and the same array, but in adults [46] (Additional file 1 - Figure S1).

CpG sites with the highest inter-individual variability were enriched for CpG shores, whereas CpG sites with high intra-individual variability were enriched in open sea areas. We further aimed to evaluate the importance of blood cellular composition in blood DNA methylation variability. For this, we applied the same model for variance partition but without residualizing the effect of blood cell proportions (Additional file 1 - Figure S2). When these were added as explanatory variables, differences in immune cell type composition explained a median of 14.0% of the intra-individual variance (Q1: 6.0%; Q3: 34.8%).

### Gene expression

Intra-individual variability explained the majority of the variance in most of the transcript clusters—groups of probes that define the expression of a gene (median: 93%; Q1: 78%; Q3: 100%).

### MiRNAs

MiRNAs presented a variance partition pattern similar to the gene expression, although not as pronounced. Intra-individual variability was predominant for most of the miRNAs (median: 65.2%; Q1: 51.8%; Q1: 81.4%).

### Proteins

Proteins presented large heterogeneity as well (median cohort variability median: 15.7%; Q1: 4.5%; Q3: 35.3%; median intra-individual variability: 60%; Q1: 48.8%; Q3: 66.3%;). For instance, the variability of C-reactive protein (CRP) was largely explained by intra-individual variability (87.2%), while the variability of the epidermal growth factor protein (EGF) was attributed to cohort by 55%.

### Serum and urine metabolites

The serum metabolome presented, in average, the highest inter-individual variability (median: 43.4%; Q1: 31.1%; Q3: 53.7%), and the lowest intra-individual variability (median: 50.7%; Q1: 41.3%; Q3: 60.3%). Urine metabolites also presented a relatively high median inter-individual variability (median: 28.82%; Q1: 16%; Q3: 40.2%), compared to other omics.

### Gaussian graphical model networks identify few CpG-metabolite change dependencies

**Fig. 3:**
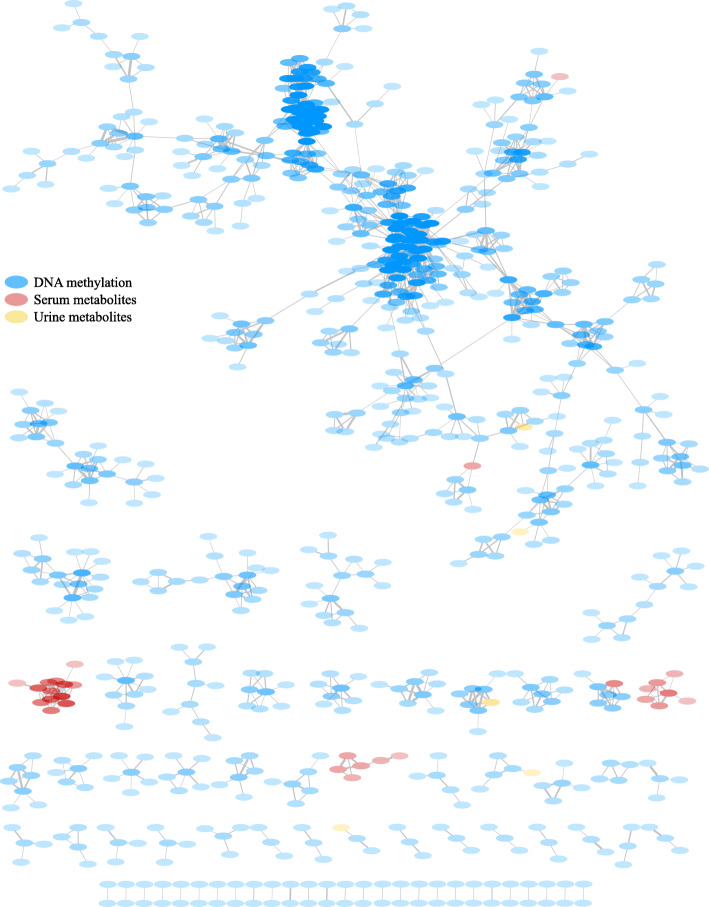
Network representation of the Gaussian graphical model (GGM) of the DNA methylome, proteins, serum and urine metabolites with high intra-individual variability measured in 157 children from five European countries. Blue nodes represent CpG sites. Red and yellow nodes represent serum and urine metabolites, respectively. The opacity of the nodes is dependent on their degree (number of edges connecting a particular feature). The edge thickness was weighted based on the partial correlation coefficients (PCCs) obtained from the GGM

**Fig. 4:**
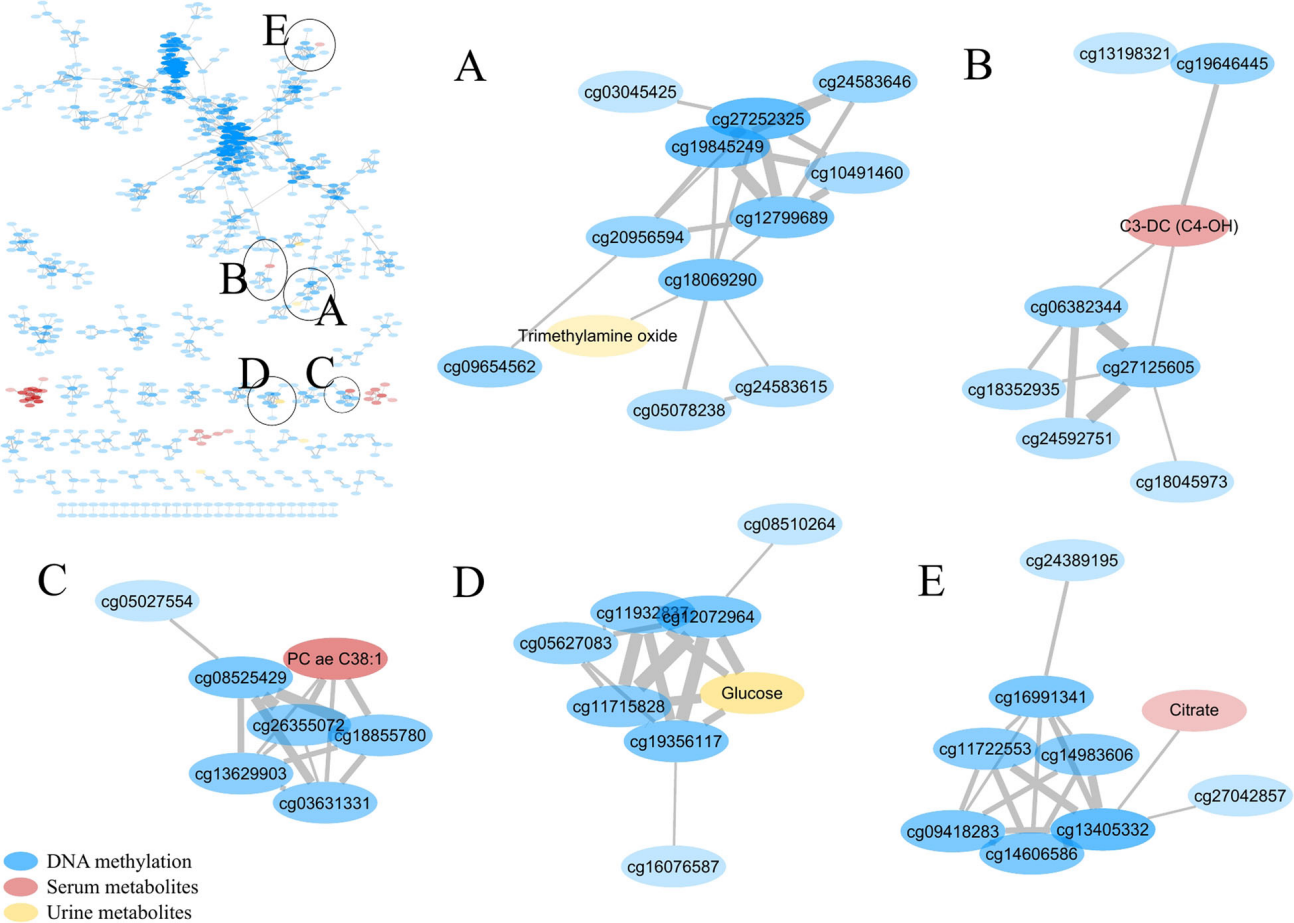
Main connected components of the Gaussian graphical model (GGM) network that involve direct associations between features from different omics layers. Blue nodes represent CpG sites. Red and yellow nodes represent serum and urine metabolites, respectively. The opacity of the nodes is dependent on their degree (number of edges connecting a particular feature). The edge thickness was weighted based on the partial correlation coefficients (PCCs) obtained from the GGM

Our multi-omics study design also allowed us to analyse co-dependencies in the variability of biological features across the different molecular layers. After applying GGM on the delta matrix (e.g. correlations on the change in omics features between visits, see the “Methods” section [54–58]), we found 70 connected components and a total of 755 nodes and 1781 undirected edges (FDR < 0.1, Fig. 3). Edges were weighted using partial correlation coefficients [PCCs ranged from 0.003 to 0.007, p values ranged from 2.22 × 10^−16^ to 1.28 × 10^−7^]. The largest connected component contained 409 nodes mainly formed by CpG sites (99%), plus 69 smaller connected components that contained from 26 to 2 nodes. Most connected components (88.5%) were formed by features from the same omics layer, including three formed exclusively by serum metabolites as follow: (1) amino-acids (Arg, Phe, Trp, Met, Met.SO, Tyr and His), (2) carnitines and (3) phosphatidylcholines (PCs). Proteins included in the model (P=9) did not show any significant partial correlation to other features. Among the connected components composed by features from different omics layers, all consisted of one serum or urine metabolite directly correlated to a group of CpGs, indicating CpG-metabolite change dependencies. These metabolites included trimethylamine oxide (TMAO), carnitine C3-DC (C4-OH), PC ae C38:1, glucose and citrate (Fig. 4A–E, respectively). CpGs cg16076587 and cg08510264 present in the same connected component as glucose are annotated to *INPP5A* (*Inositol polyphosphate-5-phosphatase A*) and *IRS2* (*Insulin receptor substrate 2*) genes, respectively.

### Biological traits can help to interpret some of the omics variability, especially in the serum metabolome

We further aimed to evaluate the association of omics variability with several anthropometric and dietary traits. Overall, the inclusion of explanatory variables accounted for up to 9% of the serum metabolite inter-individual variability (change from a median of 43% to 34% with additional explanatory variables) and up to 3–4% (median change) of the intra-individual variability in the gene expression, miRNA and proteins (Additional file 1 - Table S1). Percentage of omics features explained by each explanatory variable per omics dataset, considering three different thresholds: ≥ 1%, ≥2% and ≥ 5% of variance explained are also provided (Additional file 1 – Table S2).

**Fig. 5:**
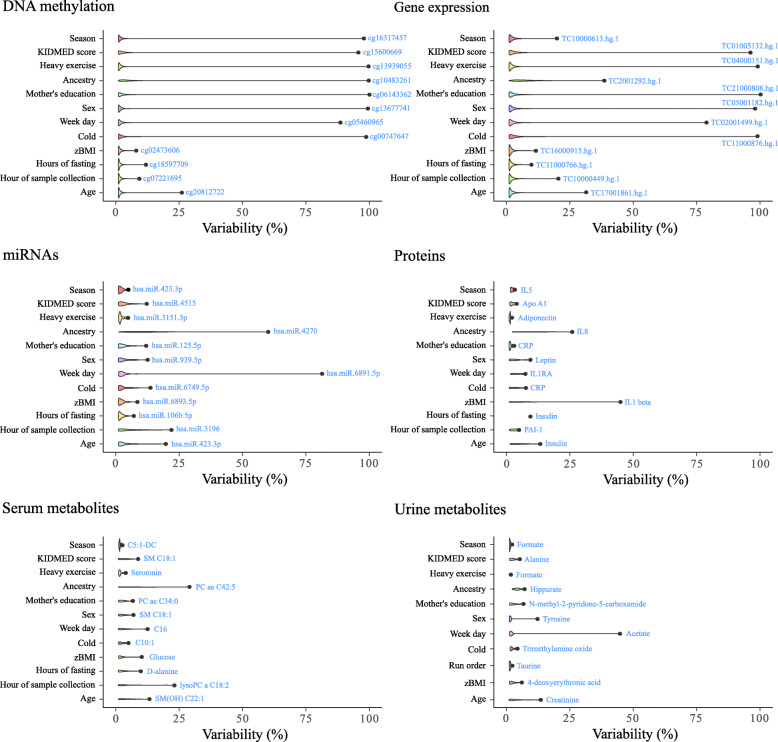
Violin plots showing multi-omics variability decomposed by biological traits and sample collection parameters measured in the study. Labels correspond to omics features mostly explained by each variable. Abbreviations. Proteome: IL: interleukin; Apo A1: apolipoprotein A1; RA: receptor antagonist; CRP: c-reactive protein. Serum metabolome: C: acylcarnitine; SM: sphingomyelin; PC: phosphatidylcholine; lysoPC: lysophosphatidylcholine

On average, for all the omics, intra-individual variability was negligibly affected by the inclusion of the explanatory variables. However, these variables explained a large percentage of variance in some particular features (Fig. 5) as described further below. Considering all the omics features as a whole, we identified that age, zBMI and hour of sample collection had a major effect on feature variability.

Generally, DNA methylation was poorly explained by the explanatory variables: only 3% of the CpGs had more than 2% of the variance explained by individual traits; the KIDMED score explained the most variability (≥ 2% of the variance in 9.6% of CpGs).

In gene expression, age, maternal education, KIDMED score, week day and hour of sample collection were responsible for at least 2% of the variance in more than 5% of the transcripts each, with the KIDMED score showing the largest influence. MiRNAs were mainly influenced by the hour of sample collection, age, ancestry, and KIDMED score. Concretely, ancestry and hour of sample collection explained more than 5% of the variance in 4.6% and 8.7% of the miRNAs, respectively. Furthermore, the KIDMED score explained more than 2% of variance in 10.6 % of miRNAs.

Proteins were mostly influenced by zBMI, ancestry, and age, where each trait explained more than 5% of the variance in 22.2%, 16.7% and 16.7% of the proteins, respectively. Among the proteins largely explained by zBMI were leptin and insulin. Similarly, these same proteins showed sex differences, found in higher concentrations in females. Ancestry explained 25–12% of the variability in these proteins (in order of magnitude): interleukin (IL)8, TNF alfa, BAFF, insulin and HGF (see Additional file 2). Insulin appeared to be the only protein significatively influenced by the hours of fasting and CRP was associated with having a cold.

Serum metabolites were mostly affected by ancestry and age, explaining more than 5% of the variance in 24.3% and 16.4% of the metabolites, respectively. By contrast, ancestry and age explained more than 5% of variance in just 2.3% and 9.1% of the urine metabolites, respectively. Our results showed that time to last meal explained more than 2% of the variance in only a 10.7% of the serum metabolites. Among the metabolites most influenced by sex, BMI and hours of fasting, we found sphingomyelin C18:1 and tyrosine for sex; glucose and 4-deoxyerythronic acid for BMI z-score; and alanine for hours of fasting.

## Discussion

The current study offers a multi-omics perspective of medium-term omics variability in childhood. We modelled the variability of 6 different types of omics data (blood DNA methylation, gene expression, miRNA, proteome, serum and urine metabolomes) for 156 children from five European countries at two time points with a 6-month interval, and found a large range of intra- and inter-individual variability between and within each omics profiles. We pointed out that overall intra-individual variation accounted for the largest part of the total variation in all omics. While DNA methylation and serum metabolites exhibited stronger stability over time for many features (median inter-individual variability: 37.6 and 43.4%, respectively), gene expression was the less stable omics profile in average (median inter-individual variability: 6.6%) and proteins and urinary metabolites were somewhat in the middle, with strong heterogeneity between features. Consequently, DNA methylation and serum metabolites (targeted assay) will better inform epidemiological studies that rely on single measurements to compare individuals in the search of biomarkers, whereas less stable omics profiles such as gene expression will give more reliable information to studies that assess individual trajectories over time (multiple time point measurements). We evidenced that variability of omics features comes from several sources. Besides technical or analytical variability, which we tried to control for, we identified physiological patterns in intra-individual variability through inter-omics network analysis. In all omics profiles, features with high inter-individual variability were identified, which can be ascribed to biological (between individuals) variability. While it is always preferable to adhere to standard sample collection conditions, this is not always possible, and omics features with substantial biological variation are potentially robust enough to yield meaningful findings in spite of collection inconsistencies. The small proportions of variability attributed to cohort demonstrated that standardized sample collection protocols can produce robust results in large-scale omics studies across different countries. Biological traits and sample collection variables, easy to collect in cohort studies, might help to account for the unwanted variability, in particular for metabolomics.

We found, in the case of the methylome, that the most stable CpG sites were enriched in functional methylation regions of the genome. CpG sites with the highest inter-individual variability were enriched for CpG shores, which are regions 0–2 kb from CpG islands (CpG rich regions) [33]. On the other hand, CpG sites with high intra-individual variability were enriched in open sea areas, which are isolated CpG sites in the genome that have been linked to chromosomal instability and loss of imprinting [33, 34]. Recent studies point out that phenotypically relevant CpGs tend to be located in CpGs shores [13, 16]. These CpGs with high inter-individual variability are especially relevant for large-scale epidemiological studies since these probes could be used as reliable biomarker candidates [16]. Our results reinforce that strong methylation differences between individuals already appear in childhood as previously reported [18], even in our population of similar European ancestry children. On the other hand, gene expression may require repeat sampling to account for intra-individual and technical variability, in order to generate stable enough markers to be deployed in epidemiological studies. Previous studies in healthy individuals show that gene expression profiles are mostly stable and repeatable in the short/medium term (< 5% of transcripts with high intra-individual variability) [20–22, 24]. Our results, in contrast, attributed the majority of the variation to intra-individual effects, potentially due to RNA quality that might differ between visits A and B. Effort has to be put on the initial sample preparation (DNA, RNA extraction and quality) and its harmonization across different centres or time point collection, since it strongly determines the quality of omics measurements and might hinder real biological response. To our knowledge, only two previous studies have estimated miRNA variability in terms of intra- and inter-individual effects, but these studied a longer time span (5 years) [26] or a daily time span (48 h) in cerebrospinal fluid [27]. Despite these differences in study design, both studies agree that there is diversity within the microRNome in terms of stability of its features. Previous proteome studies in healthy human volunteers have demonstrated moderate inter-individual variability (CV ranging from 30% to 50%) [35, 36], similar to the urine proteome in seven adult donors [37]. A study that estimated intra-individual variation of plasma adipokines concluded that they may be useful biomarkers of inflammation in population-based studies of obesity-related disease due to their stability over time [38]. These corroborate our results for IL-1β, IL-6, IL-8, leptin, adiponectin, hepatocyte growth factor (HGF) and CRP which presented the highest stability in our study. Finally, metabolomics studies comparing serum and urine metabolomes corroborate in children and adult populations strongly corroborate our findings that the serum metabolome is more stable and captures more inter-individual specific variance, compared to the urine metabolome [39–43].

Biological traits, such as body weight, sex and age, accounted for inter-individual variability in gene expression, similar to results obtained by Hughes et al. (2015) [25], where roughly 2% of total gene expression variation was explained by these traits in placenta samples. Age and hour of sample collection have also been found to be significant sources of variation in blood gene expression patterns of healthy individuals [60]. Previous studies on miRNA variability identified the strong influence of age and sample storage time [15, 23], but the time of the day or dietary intake has not been previously studied to our knowledge. Among the proteins largely explained by zBMI were leptin and insulin, hormones related to food intake and fat storage [61] and the pro-inflammatory cytokines IL-6 and IL-1 beta, both known to be elevated in subjects with obesity or with serum lipid concentrations abnormalities, leading to a state of chronic inflammation [62]. Similarly, these same proteins showed sex differences, in particular leptin and IL-1 beta, known to be higher in females and resulting in a higher risk to develop obesity than males [63]. Interestingly, circulating IL-8, a pro-inflammatory cytokine, was strongly influenced by ancestry (6% of children had Pakistani ancestry) in our cohorts (25% of variance explained). IL-8 was previously found to be influenced by genetic polymorphisms in an eastern Indian population, potentially driving individual variations in the host’s immune response, in particular to infectious diseases [64]. Intra-individual factors such as hours of fasting and having a cold gave the expected results: insulin appeared to be the only protein significatively influenced by the hours of fasting and CRP was associated with having a cold. This is in line with a previous study that considered the fasting/postprandial state of the samples and revealed that, on average, it explained less than 2% of the total variance [41]. Associations of serum and urine metabolites with sex, BMI and hours of fasting (only for serum) was also previously reported in the cross-sectional study of 1300 HELIX children [39]. Interestingly, high levels of 4-deoxyerythronic acid in children, found correlated to higher BMI in our study, have been previously related to early-onset type I diabetes although further understanding of its metabolism is required [65].

A few recent studies have reported changes in multiple omics profiles in clinical settings and after dietary wellness coaching intervention or physical activity [30, 66, 67], revealing omics signatures that may serve as potential diagnostic markers. However, variability of omics profiles in healthy population and “normal” living conditions remains under-studied. Large observational studies would benefit enormously from this information, because it allows the interpretation of changes that do not match a pattern. This is especially important for early-life studies that focus on the origin of diseases in children because omics profiles are able to capture very early and subtle molecular responses, even before physiological manifestations appear. Variations in omics profiles due to factors such as BMI, age, physical activity, fasting time and sampling conditions need to be well characterized in order to interpret subtle changes related to environmental exposures such as air pollution or endocrine-disrupting chemicals, the ultimate goal of recent exposome initiatives [60]. Omics measurements in our study are of great clinical relevance as they provide the basis for the discovery of new biomarkers of multiple medical conditions such as cancer, heart disease, neurological disorders or inflammatory diseases [61–64]. Here, we provide insight into which omics features are stable within individuals and demonstrate sufficient inter-individual variation in order to reduce chance findings when conducting epidemiological studies with follow-up for disease outcomes [16].

We emphasize the fact that children from five European countries took part in the study. The multiple locations of our participants allow to generalize the influence of factors such as lifestyle habits, seasonal influences and maternal education, which vary greatly between countries/cultures and not many studies consider.

A major strength of our study lies in the cross-omics approach. We created a network of interacting omics features of four different omics layers (DNA methylome, proteome, serum and urine metabolomes) using Gaussian graphical models (GGMs). GGMs circumvent the selection of indirect associations that usually appear between omics measurements, as Pearson correlations are generally high in these data. GGMs are based on partial correlation coefficients and provide an estimate of conditional dependencies between variables, elucidating direct associations [54]. This enabled us to obtain a holistic view of the biological significance of our results. By studying the interaction of multiple omics features, we exploited the data to their full potential for further disease prediction and prevention studies [56]. Moreover, it allowed us to go a step further by identifying interactions of features across omics layers, which is the main goal of the recent discipline *interactome* within systems biology. Despite not finding many dependencies across omics, a few metabolites were related to groups of CpGs. These included TMAO, a compound generated by the gut microbiota from diet-derived components, hence with high variability and strongly determined by diet, gut microbial flora and drug administration [65]. Elevated levels of TMAO positively correlate to cardiovascular disease through the development of atherosclerosis in previous studies [68–73]. Variation in four CpGs was related to glucose in urine (pool morning and night urine), which level depends on lifestyle factors like diet and exercise behaviours and its dysregulation related to obesity and type 2 diabetes (T2D) in children [74]. Interestingly, two of these CpGs are within genes related to obesity and T2D [53]. This supports previous studies on GGMs that show their ability to reconstruct metabolic pathways [54, 75, 76], including in this case across omics.

Our study had some limitations. In the first place, we studied omics variability in 156 children and a larger population size would have provided greater statistical power to our model. However, this sample size allowed us to analyse 6 different molecular layers in the same individual and at the same time-point, twice, data rarely obtained in the past. Our study used targeted approaches to measure the proteome and the serum metabolome profiles (semi-targeted for urine). While this approach provided reduced biological coverage, we are confident that this insured reliable annotation and quantification of markers. In contrast, we report high intra-individual effects in a large proportion of genome-wide omics features that are potentially due to technical variability. Despite considering quality control parameters (e.g. filtering for call rate) in order to minimize this effect, we note that precision in the intra-individual variability apportionments can be strengthened by adding technical replicates. This, together with increasing the number of individuals, would be the ideal way to strengthen the biological signal. Moreover, despite having measured many biological traits in the children under study, there are relevant missing variables, such as recent dietary intake in the last 24 h, which is expected to influence the variability of omics such as the urine metabolome. It would be interesting to study if and how other factors account for variability within omics. Our study could also benefit from a more exhaustive collection of repeated samples across short periods of time. For example, having morning and night samples in the same individual at different time points, as done previously for metabolomics [77, 78] or miRNAs [27].

## Conclusions

We assessed omics profiles variability over the medium-term in child cohorts from the general population, using a multi-omics approach. We found large heterogeneity within and between omics profiles. Intra-individual variability presented the highest median variability in all cases. The cross-omics analysis we performed provides global insights into how different omics features vary over time, within and between individuals and among cohorts from different countries. This study thereby provides a valuable framework for future epidemiological studies that aim to detect omics signatures linked to disease, environmental exposures or both.

## Supplementary Information

**Additional file 1.** Supplementary tables, figures and R code. This file contains supplementary methods, tables and figures summarizing the results of the variance partition models. It also contains the code used in R to perform variance partition analyses and Gaussian graphical models.

**Additional file 2.** Results of the variance explained by each explanatory variable in each omics feature. This file contains, for each omics layer (one per sheet), the percentage of variance explained by each explanatory variable in each omics feature.

## Data Availability

The summarized results generated during the current study are available in supplementary material. The raw data supporting the current study are available from the corresponding author on request subject to ethical and legislative review.

## Abbreviations

BMI: Body mass index
CRP: C-reactive protein
CV: Coefficient of variation
FDR: False discovery rate
GGM: Gaussian graphical model
HELIX: Human Early Life Exposome
ICC: Interclass correlation coefficient
IL: Interleukin
KIDMED score: Mediterranean diet quality index
miRNA: MicroRNA
PC: Phosphatidylcholine
PCCs: Partial correlation coefficients
Q1, 2, 3, 4: Quartile 1, 2, 3, 4
QC: Quality control
SD: Standard deviation
SVA: Surrogate variable analysis
TC: Transcript cluster
TMAO: Trimethylamine oxide
