# Supplementary material for: Variability of multi-omics profiles in a population-based child cohort
Source: BMC Med. 2021 Jul 22;19:166. doi: 10.1186/s12916-021-02027-z (PMC8296694; doi:10.1186/s12916-021-02027-z)
Supplement: Supplementary file 1 — Additional file 1. Supplementary tables, figures and R code. This file contains supplementary methods, tables and figures summarizing the results of the variance partition models. It also contains the code used in R to perform variance partition analyses and Gaussian graphical models. [file 12916_2021_2027_MOESM1_ESM.docx]

**Variability of multi-omics profiles in a population-based child cohort**

Marta Gallego-Paüls^1,2,3^, Carles Heráandez-Ferrer^1,2,3^, Mariona Bustamante^1,2,3,4^, Xavier Basagaña^1,2,3^_,_ Jose Barrera-Gómez^1,2,3^, Chung-Ho E. Lau^5,6^, Alexandros P. Siskos^7^, Marta Vives-Usano^1,2,3,4^, Carlos Ruiz-Arenas^1,2,3^, John Wright^8^, Remy Slama^9^, Barbara Heude^10^, Maribel Casas^1,2,3^, Regina Grazuleviciene^11^, Leda Chatzi^12^, Eva Borràs^2,4^, Eduard Sabidó^2,4^, Ángel Carracedo^13,14^, Xavier Estivill^4^, Jose Urquiza^1,2,3^, Muireann Coen^6,15^, Hector C. Keun^7^, Juan R. González^1,2,3^, Martine Vrijheid^1,2,3^, Léa Maitre^1,2,3*^

# **Additional file 1**

## Supplementary Methods

### Laboratory processing of omics signatures, quality control and normalization

#### Blood DNA methylation

DNA was obtained from buffy coat collected in EDTA tubes at age 7‐9y. Briefly, DNA was extracted using the Chemagen kit (Perkin Elmer) in batches of 12 samples. Samples were extracted by cohort. DNA concentration was determined in a NanoDrop 1000 UV‐Vis Spectrophotometer (ThermoScientific) and with Quant‐iT™ PicoGreen® dsDNA Assay Kit (Life Technologies).

DNA methylation was assessed with the Infinium HumanMethylation450 beadchip from Illumina, following manufacturer’s protocol. Briefly, 700 ng of DNA were bisulfiteconverted using the EZ 96‐DNA methylation kit following the manufacturer’s standard protocol, and DNA methylation measured using the Infinium protocol. A HapMap sample HELIX – Methylome QC was included in each plate. In addition, 24 HELIX inter‐plate duplicates were included. Samples were randomized considering cohort, sex and panel. Samples from the panel study (same subject) were processed in the same plate and array. Two samples were repeated due to their overall low quality.

DNA methylation data was pre‐processed using the minfi package[39]). We increased the stringency of the detection p‐value threshold to <10E‐16, and probes not reaching a 98% call rate were excluded [40]. Two samples were filtered due to overall quality: one had a call rate <98% and the other did not pass QC parameters of the MethylAid package [41]. Then, data was normalized with the functional normalization method, which also includes Noob background subtraction and dye‐bias correction [42]. After that, several quality control checks were performed. First, we checked sex consistency using the shinyMethyl package [43] and two samples were excluded. Genetic consistency of duplicates and samples from the same participant was checked with the 450k genotypes. In addition, genetic consistency was evaluated in those samples that had GWAS data and two of them were excluded. Principal component analysis showed no differential clusters, however a degree of grouping within the cluster was observed for sex and cohort and for some technical variables. Finally, duplicated samples and HapMap samples were removed as well as control probes, probes designed to detect SNPs and probes to measures methylation levels at non‐CpG sites. The final dataset consisted of 149 subjects and 386,518 probes after filtering of probes with SNPs, probes that cross‐hybridize and probes in sexual chromosomes. Residuals of surrogate variables estimated with the surrogate variable method (SVA) were calculated protecting for cohort, sex and age, and used in for statistical analysis of DNA methylation [44,45].

Further filtering of probes was applied based on their interclass correlation coefficient (ICC) according to previous publications with technical replicates [46] that suggest that ICC values for technical replicates can be used as a measure of reliability for methylation measurements. Supplementary Figure 2 from Additional File 1 shows the correlation between the intra-individual variability and the ICC value for the 386,518 probes. We could appreciate that the most variable (highest intra-individual variability) CpG sites were indeed those with lower ICC. Concretely, we observed a gradient change in the regression at 62.5% ICC. Therefore, we decided to filter out methylation probes not reaching this ICC value in order to increase the reliability of the biological interpretation of the results in our study.

**Gene expression**

RNA was extracted using the MagMAX for Stabilized Blood Tubes RNA Isolation Kit (TermoFisher). The quality of RNA was evaluated with a 2100 Bioanalyzer (Agilent) and the concentration with a NanoDrop 1000 UV‐Vis Spectrophotometer. Samples classified as good RNA quality had a RIN >5, a similar RNA integrity pattern in the visual inspection (bioanalyzer) and a concentration >10 ng/ul.

Gene expression, including coding and non‐coding transcripts was assessed with the Affymetrix Human Transcriptome Array 2.0 ST arrays (HTA 2.0). Samples were processed at the University of Santiago de Compostela (USC). In each round, several batches of 24‐48 samples were processed. Samples were randomized within each batch considering sex and cohort. Samples from the same subject (panel study) were processed in the same batch. Raw data were extracted with the AGCC software (Affymetrix) and stored into CEL files.

Data was normalized by Affymetrix with the GCCN (SST‐RMA) algorithm at the gene and transcript level. Annotation to transcripts clusters was done with the ExpressionConsole software using the HTA‐2_0 Transcript Cluster Annotations Release na36. After normalization several quality control checks were performed and four samples with discordant sex were excluded.

Control probes and probes in sexual chromosomes or probes without chromosome information were excluded. Probes with a DABG (Detected Above Background) p value < 0.05 were considered to have an expression level different from the background, and they were defined as detected. Gene expression values were log2 transformed. Although we only observed one cluster of samples in the Principal Component Analysis (PCA), there was some grouping of samples within the cluster by cohort and by technical variables. To control for this potential confounding by technical bias and cell type proportions, the residuals of surrogate variables estimated on gene expression with the SmartSVA method protecting for cohort, sex and age [7] were obtained and use for statistical analysis. The final dataset consisted of 45,438 probes. In addition, probes with a call rate <25% were excluded from the analysis in order to minimize overestimation of intra-individual variability by technical effects (Supplementary Figure 3 - Additional File 1).

**miRNAs**

Expression miRNA levels of samples with good RNA quality were analysed using the SurePrint Human miRNA Microarray rel. 21 (Agilent) at the Genomics Core facility at the Centre for Genomics Regulation (CRG). Samples were randomized by sex and cohort and samples of the same subject (panel study) were processed in the same batch and array. Batches consisted of 24 samples which were hybridized onto 3 slides (8 samples per slide). Raw data and GeneView files were extracted with the Feature Extraction software (Agilent). Samples that did not pass the laboratory quality control parameters were repeated. miRNAs were annotated with the Annotation_70156 version from Agilent and with additional information from mirBase v21 (<http://www.mirbase.org/>).

We performed an initial quality control which included: check of the Agilent quality control parameters, check of the number of miRNAs in duplicated samples, and calculation of the sample and miRNA call rate. Agilent considers that miRNAs are not detected when the expression signal is not different from the background or the standard error of the different probes is >3 times higher than the expression signal. The least variant set (LVS) method was applied for the normalization of the samples that passed the quality control. For the identification of housekeeping miRNAs with the LVS method, a random sample of 50 HELIX samples was used. After normalization, miRNAs in sexual chromosomes or not annotated were filtered out. The final dataset consisted of 1,117 miRNAs. MiRNA expression values were log2 transformed. To control for potential confounding by technical bias and cell type proportions, the surrogate variable analysis (SVA) method [44] was applied, protecting for cohort, sex and age, during the statistical analysis and the residuals of the correction were used for statistical analysis of miRNAs. Finally, miRNAs with call rates <25% were excluded from the analysis to ensure minimization of technical variability within intra-individual variability (Supplementary Figure 4 - Additional File 1).

**Proteins**

A set of 43 proteins were selected a priori based on the literature and on the Luminex kits commercially available from Life Technologies and Millipore. Three kits were selected for the subsequent analyses, which assessed a total 50 measurements: Cytokines 30-plex (Cat #. LHC6003M), Apoliprotein 5-plex (LHP0001M) and Adipokine 15-plex (LHC0017M). samples from HELIX cohorts were analysed in the CRG/UPF Proteomics Unit with the xMAP and Luminex system and following the manufacturer’s protocol. The experimental design was planned to control for batch effects and confounding variables. All samples were randomized and blocked by cohort prior measurement to ensure a representation of each cohort in each measurement plate (batch). No distinction was made by panel nor by gender. For protein quantification, an 8-point calibration curve per plate was performed with protein standards provided in the Luminex kit and following the procedures described in the standard procedures described by the vendor.

Raw intensities obtained with the xMAP and Luminex system for each plasma sample were converted to ng/ml (5-plex kit: adiponectine, CRP, APO-A1, APO-B, APO-E) and to pg/ml (15 and 30-plex kits) using the calculated standard curves of each plate and accounting for the dilutions that were made prior measurement. The % of coefficients of variation (% CV) for each protein estimated by plate and then averaged ranged from 3.42% to 36%. For each protein, the limit of detection (LOD) was determined and the lower and upper quantification limits (LOQ1 and LOQ2, respectively) were obtained from the calibration curves. Seven proteins were removed because they had <30% of measurements in the linear range (3 of them had values <LOD or <LOQ1: IL7, VEGF, GMCSF; and 4 of them had values >LOQ2: Lipocalin2, RANTES, Resistin, SAA). Seven proteins were measured in two different plex and the measure with lower quality was excluded from the analysis. For the 36 proteins that passed the QC, data was log2 transformed to reach normal distribution. Then, the plate batch effect was corrected by subtracting for each individual and each protein the difference between the overall protein average minus the plate specific protein average. Finally, values below LOQ1 and above LOQ2 were imputed using a truncated normal distribution implemented in the truncdist R package. This method performs imputation of missing data using random draws from a truncated distribution with parameters estimated using data in the linear range of each protein (e.g between LOQ1 and LOQ2). The final dataset contained the log2 transformed, imputed and normalized levels for 36 proteins.

**Serum metabolites**

Serum metabolites were analysed using metabolomics AbsoluteIDQ^TM^ p180 Kit (BIOCRATES Life Sciences AG) [78]. The kit allows the targeted analysis of 188 metabolites in the metabolite classes of amino acids, biogenic amines, acylcarnitines, glycerophospholipids, sphingolipids and sum of hexoses, covering a wide range of analytes and metabolic pathways in one targeted assay. Batches of samples were fully randomised to prevent potential analytical biases that would impact on subsequent data processing and statistical analysis.

For the LC-MS/MS assay, the metabolites were quantified by stable isotope dilution and seven-point calibration curves. For the FIA-MS/MS assay, metabolite concentrations were calculated using a one-point internal standard calibration, and are also isotope corrected. Metabolites were quantified (results shown in micromolar (μΜ) concentration units) according to the manufacturer’s protocol using the MetIDQ™ Boron software for targeted metabolomics data processing and management. Blank PBS (phosphate-buffered saline) samples (3 replicates) were used for the calculation of the limits of detection (LOD). The median value of the PBS samples on the plate was calculated as approximation of the background noise per metabolite, and 3 times this value was calculated as the LOD, per metabolite, per batch. Also in every analytical batch, three sets of quality control samples were included, the NIST SRM 1950 plasma reference material (in 4 replicates), a commercial available serum QC material (CQC in 2 replicates, SeraLab, S-123-M-27485) and the QCs provided by the manufacturer in three concentration levels. The NIST SRM 1950 reference was used as the main quality control sample for the LC-MS/MS analysis. Coefficients of variation (CVs) for each metabolite were calculated based on the NIST SRM 1950. To avoid imputation during subsequent statistical analysis recorded values below the limits of detection (BLD) were not excluded from the analysis. Data was log2 transformed to reach normal distribution.

Data for 188 metabolites were acquired with the LC/FIA-MS/MS targeted metabolomics protocol. Of the 188 detected metabolites, 11 metabolites were excluded from subsequent statistical analysis that met both the following two criteria: i) %CV > 30% AND ii) % BLD + zeros >30 %. These are 11 metabolites (Carnosine, DOPA, Dopamine, nitro-Tyr, c4-OH-pro, PEA, Histamine, C3:1, C18:1-OH, PC aa C26:0, SM C22:3). Therefore, the final dataset of the serum metabolome consisted of a total of 177 metabolites.

**Urine metabolites**

Proton Nuclear Magnetic Resonance Spectroscopy (^1^H NMR) were was used to profile urine metabolites [78]. Urine metabolic profiles were analysed on a Bruker Avance III spectrometer operating at 14.1 Tesla (600MHz ^1^H) NMR spectrometer at Imperial College London (ICL) in the final quarter of 2015. All samples and batching were fully randomised to prevent potential bias in the analytical run from impacting on subsequent data processing/analysis. Aliquots of the study pooled quality control (QC) sample were used to monitor analytical performance throughout the run and were analysed at an interval of every 23 samples (i.e. 4 QC samples per well plate). The ^1^H NMR spectra were acquired using a standard one-dimensional solvent suppression pulse sequence. We analysed the combined urine samples whenever possible. The morning and night samples were only analysed if we could not create a combine pool due to missing samples. A low coefficient of variation (CV) represents high analytical precision/stability from repeated measures. CV and % below limit of detection were characterized per metabolite and CV were below 30% for all 44 metabolites, indicating that sample run was of good analytical reproducibility. The final dataset contained log2 transformed and median fold change normalised levels for 44 urine metabolites.

### Statistical analyses – R codes

**Variance partition analysis using R-package *variancePartition [47]***

***#Example for proteome***

library('variancePartition')

#load dataset with rows containing individuals and columns containing omics measurements and metadata

load("P:/Omics_variability/Final datasets/proteome.RData")

#create matrix containing omics measurements

proteome_matrix <- data.matrix(t(proteome[,2:37]))

#create matrix containing metadata

proteome_info <- as.data.frame(proteome[,38:76])

#create an object that contains the variables to model

#FIRST MODEL, modelling the effect of the individual and the cohort, without explanatory variables

form_1 <- ~ (1|ID) + (1|cohort)

#SECOND MODEL, including explanatory variables (continuous variables should be modelled as fixed effects)

form_2 <- ~ (1|ID_individual) + (1|cohort) + age + time_sampling + hours_fasting + zBMI + (1|sex) + (1|weekday) + (1|season) + (1|ethnicity) + (1|exercise) + (1|cold) + (1|education_mother) + (1|KIDMED_score)

#run models

varPart_1 <- fitExtractVarPartModel(proteome_matrix, form_1, proteome_info )

varPart_2 <- fitExtractVarPartModel(proteome_matrix, form_2, proteome_info )

#save results in .csv file

write.csv(varPart_1, file="proteome_variancePartition_1.csv")

write.csv(varPart_2, file="proteome_variancePartition_2.csv")

**Gaussian graphical models (GGMs) using R-package *GeneNet [48]***

Previous to this: create a matrix “variables” containing all the omics measurements included in the GGM (columns) for each individual at each visit, ID_1A and ID_1B (rows).

#create a vector will all the IDs, regardless the visit.

ID <- unique(gsub("_1A","",gsub("_1B","",rownames(matrix))))

#calculate the delta matrix: change/difference between visit a and visit b for each marker, each individual

delta <- c()

for (i in 1:ncol(matrix)){

delta0 <- c()

for (k in 1:length(ID)){

delta0 <- cbind(delta0,dist(matrix[grep(ID[k],rownames(matrix)),i]))

}

delta <- rbind(delta,delta0)

}

deltat <- t(delta)

colnames(deltat) <- colnames(matrix)

#compute GGMs

pcor_omics <- ggm.estimate.pcor(deltat, method = c("dynamic"))

#estimate optimal shrinkage

edges<- network.test.edges(pcor_omics, fdr=TRUE, direct=FALSE, plot=FALSE)

#extract network containing significant associations with probability >0.95 (FDR<0.05)

net <- extract.network(edges,cutoff.ggm=0.95)

## Supplementary Results


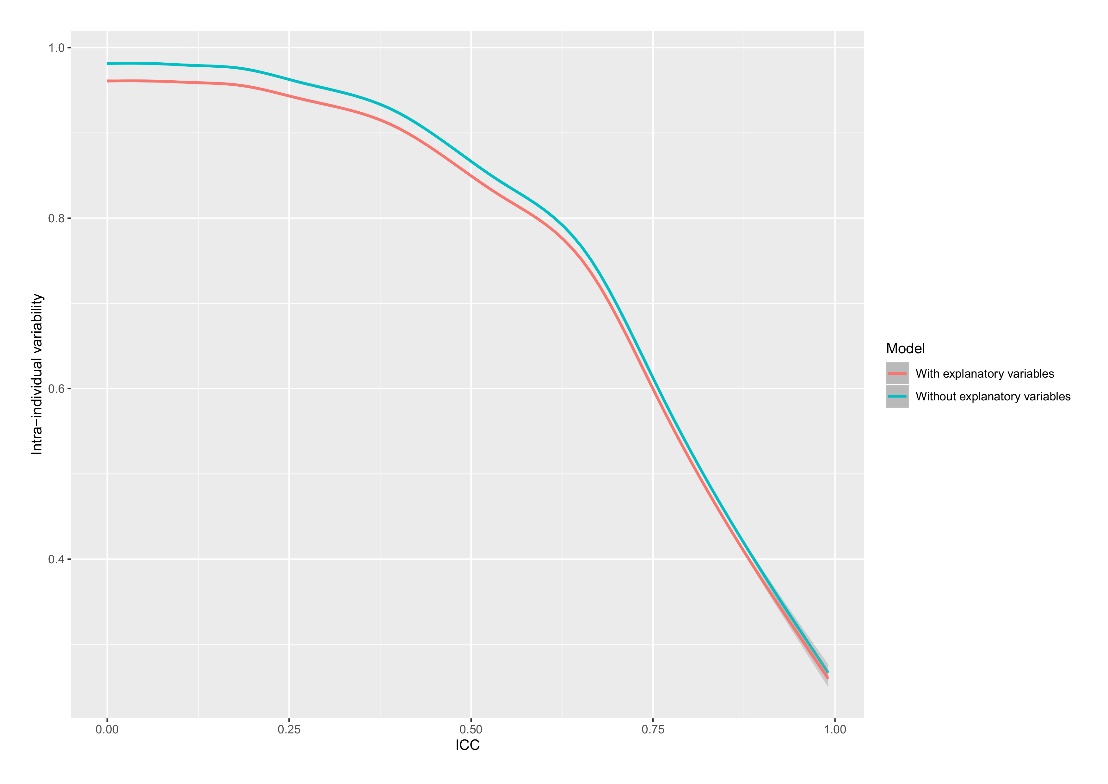


**Figure S1.** Correlation between CpGs intra-individual variability and their Interclass correlation coefficients (ICC) according to previous publications with technical replicates [8].


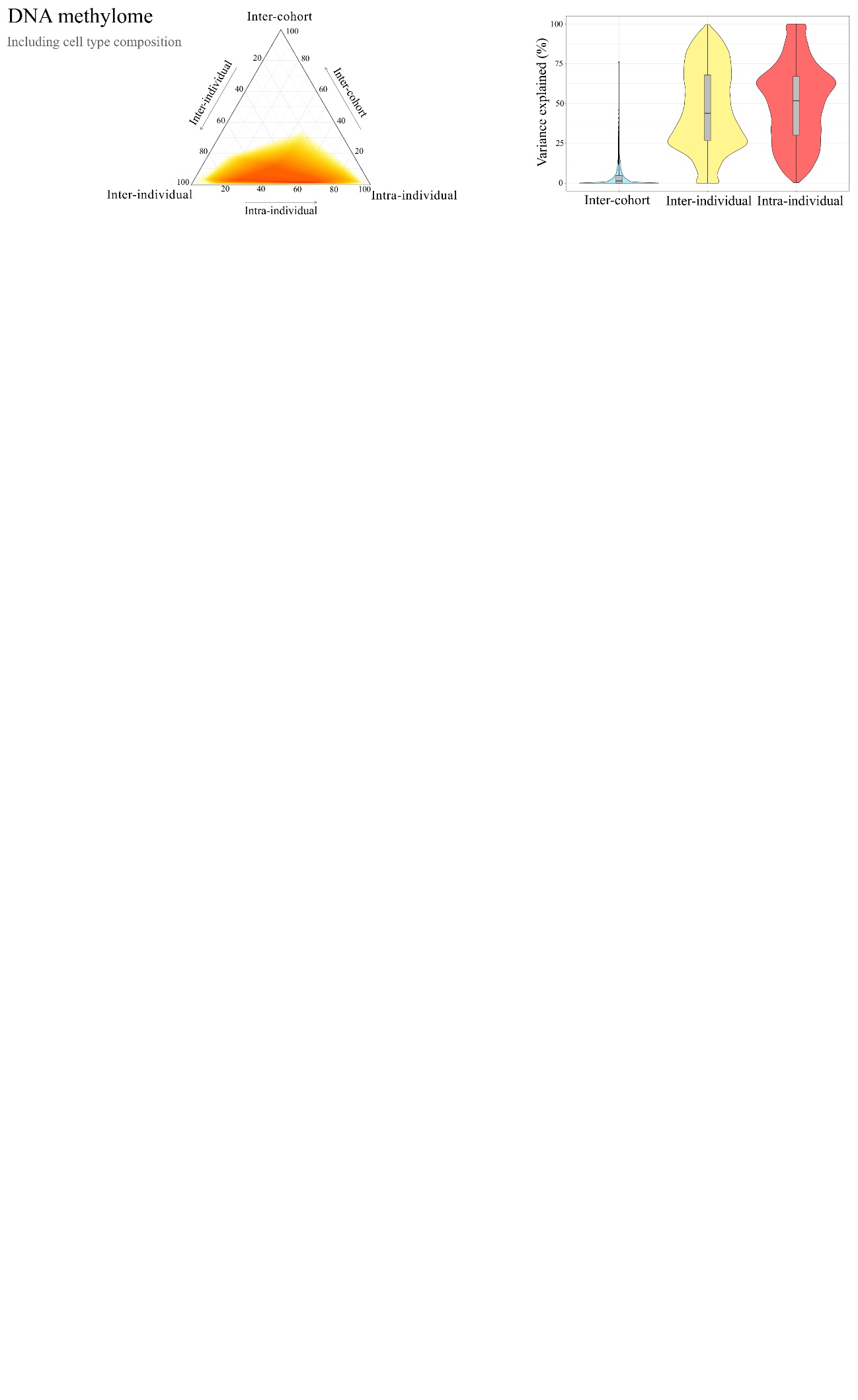
 **Figure S2.** DNA methylome variance partition model without residualizing the effect of blood cell proportions.


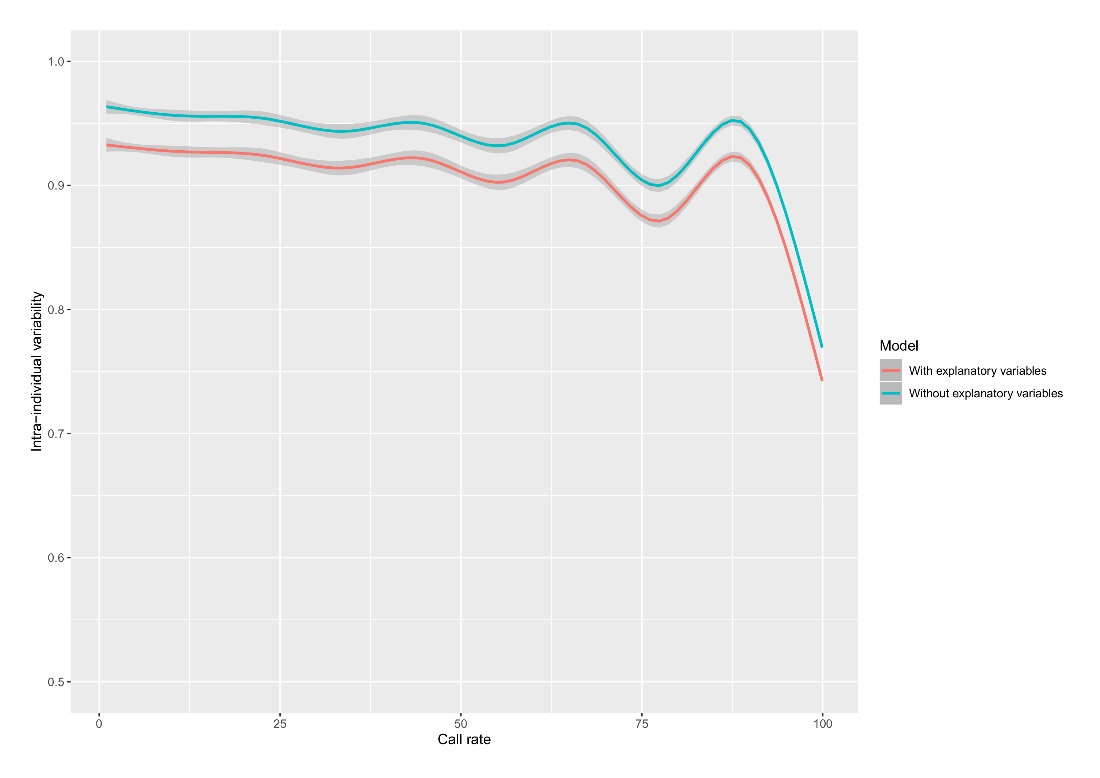


**Figure S3.** Correlation between gene expression intra-individual variability and probes call rate, as a measure of technical variability.


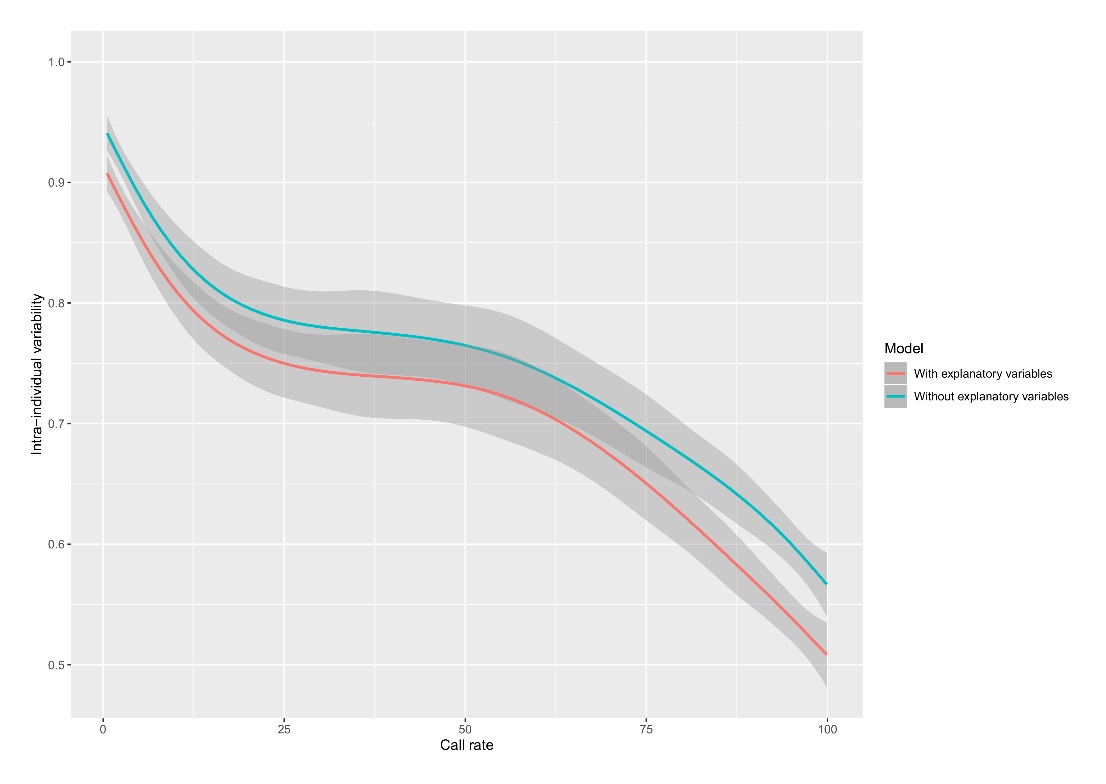


**Figure S4.** Correlation between miRNAs intra-individual variability and probes call rate, as a measure of technical variability.

**Table S1.** Descriptive of the variance partition analysis (with and without explanatory variables) performed in each omics profile.

**Table S2.** Percentage of features explained by each explanatory variable in each omics profile, considering three different thresholds: ≥1%, ≥2% and ≥5% of variance explained.
